# Effectiveness of Influenza Vaccine in Wuhan, China During the 2024–2025 Season: A Test-Negative Case–Control Study

**DOI:** 10.3390/vaccines14030243

**Published:** 2026-03-06

**Authors:** Pei Zhang, Xiaokun Yang, Banghua Chen

**Affiliations:** 1 Office of Preventive Medicine Association, Henan Center for Disease Control and Prevention, Zhengzhou 450016, China; daidaolangman@126.com; 2 Wuhan Center for Disease Control and Prevention, Wuhan 430024, China; 3 Division of Infectious Disease Control and Prevention, Chinese Center for Disease Control and Prevention (Chinese Academy of Preventive Medicine), Beijing 102206, China; yangxk@chinacdc.cn; 4 Chinese Field Epidemiology Training Program, Chinese Center for Disease Control and Prevention (Chinese Academy of Preventive Medicine), Beijing 100050, China

**Keywords:** influenza, vaccine effectiveness, test-negative design

## Abstract

**Background**: Vaccine Effectiveness (VE) provides an important indicator of vaccine performance under real-world conditions. However, evidence regarding influenza VE in Wuhan remains limited. This study applied a test-negative case–control design to estimate the effectiveness of influenza vaccination during the 2024–2025 influenza season in Wuhan. **Methods**: A test-negative case–control design was conducted among patients presenting with influenza-like illness (ILI) at outpatient and emergency departments of 41 healthcare institutions in Wuhan. All participants underwent influenza virus real-time reverse transcription polymerase chain reaction (RT-PCR) testing and were categorized as cases (RT-PCR positive) or controls (RT-PCR negative) based on laboratory results. **Results**: The analysis included 23,302 RT-PCR-confirmed influenza cases and 99,424 test-negative controls. The overall adjusted VE was 35% (95% CI: 30–40%). VE differed across age group, with higher estimates observed among adults aged 19–59 years (63%; 95% CI: 50–73%) and 60–69 years (60.7%; 95% CI: 46–72%), whereas lower effectiveness was observed among children aged 0.5–5 years (25%; 95% CI: 17–33%) and 6–18 years (25%; 95% CI: 14–36%). Similar protection was observed among individuals vaccinated in both the 2023–2024 and 2024–2025 seasons (42%; 95% CI: 28–54%) and those vaccinated in the 2024–2025 season only (40%; 95% CI: 36–45%), whereas VE was lower among individuals vaccinated only during the previous season (20%; 95% CI: 14–26%). VE also varied by vaccination timing, with the highest effectiveness observed among individuals vaccinated in November 2024 (46.1%; 95% CI: 36.4–54.6%). **Conclusions**: Influenza vaccination provided measurable protection during the 2024–2025 season in Wuhan. Greater protection was observed among individuals vaccinated in consecutive seasons or during the current season compared with those vaccinated only in the prior season. Vaccination administered in November was associated with the highest effectiveness, highlighting the importance of appropriate vaccination timing.

## 1. Introduction

Influenza is a major respiratory infection caused by influenza viruses and remains a significant global health concern across all age groups. It is estimated that influenza is responsible for approximately 500,000 deaths worldwide each year, with the vast majority occurring among adults aged ≥65 years [1,2,3,4]. Seasonal influenza vaccination is widely recognized as a key preventive strategy for reducing infection risk and limiting severe clinical outcomes, thereby alleviating the overall disease burden [5,6]. In China, influenza vaccines are not included in the national immunization program, and vaccination coverage remains relatively low, with uptake rates of 3.16% during the 2020/21 season and 2.47% during the 2021/22 season [7]. Such limited coverage may contribute to sustained transmission and a substantial public health burden. Vaccine effectiveness (VE) provides an important measure of vaccine performance under real-world conditions, particularly in the context of antigenic drift and evolving circulating influenza viruses [8]. To date, evidence regarding influenza VE in Wuhan remains limited. As a major metropolitan area with high population mobility, Wuhan requires region-specific VE evaluation to better inform local vaccination strategies. Influenza vaccine effectiveness has been shown to vary substantially across geographic regions, influenza seasons, and circulating virus strains [9,10,11,12]. Differences in demographic structure, population mobility, healthcare accessibility, and climatic factors may influence virus transmission dynamics and vaccine performance [12,13]. Several international surveillance collaborations, including the Global Influenza Vaccine Effectiveness (GIVE) network, have highlighted the importance of region-specific VE evaluation to support evidence-based vaccination policies [11,14]. Large metropolitan areas with dense populations and frequent population movement, such as Wuhan, may present unique influenza transmission patterns that differ from national or provincial averages. Therefore, local VE assessment is essential for optimizing vaccination strategies and strengthening regional influenza prevention and control programs [13]. Therefore, this study assessed influenza VE during the 2024–2025 season in Wuhan using a test-negative case–control design.

The test-negative case–control approach represents an adaptation of the conventional case–control design [15] and has become increasingly utilized in influenza vaccine effectiveness research. This methodology allows VE to be assessed throughout different phases of the influenza season and helps reduce potential bias related to healthcare-seeking behavior and diagnostic misclassification [16]. It has been incorporated into routine influenza VE surveillance programs in several countries, including the United States [9], Canada [10], and Australia [17].

## 2. Materials and Methods

### 2.1. Study Subjects

This study employed a test-negative case–control design, which is widely used for evaluating influenza vaccine effectiveness under real-world conditions [15]. In this design, patients seeking medical care for influenza-like illness (ILI) were systematically tested for influenza virus infection using RT-PCR. Individuals with laboratory-confirmed influenza were classified as cases, while those testing negative served as controls. By restricting enrollment to patients presenting with similar clinical symptoms and healthcare-seeking behavior, the test-negative design helps reduce bias associated with differential healthcare utilization [16]. To minimize potential confounding and selection bias, several measures were implemented. First, standardized ILI case definitions were applied across all participating healthcare institutions. Second, laboratory testing procedures followed uniform RT-PCR protocols recommended by the National Influenza Center to ensure consistency and diagnostic accuracy [18]. Third, vaccination status was verified through the immunization information system whenever possible to reduce recall bias. Finally, multivariable logistic regression models were used to adjust for age and sex, further reducing confounding effects. This methodological approach enhances the internal validity of VE estimation and is consistent with international influenza vaccine effectiveness monitoring practices.

Participants were enrolled from outpatient and emergency departments of 41 secondary- or tertiary-level hospitals in Wuhan that performed influenza virus RT-PCR testing (Appendix A Table A1). Based on the seasonal pattern of influenza circulation in Wuhan, the study period extended from 1 October 2024 to 30 April 2025. Eligible participants were patients aged ≥6 months who met the influenza-like illness (ILI) definition specified in the National Influenza Surveillance Technical Guidelines (2017 Edition) [18], defined as fever (body temperature ≥38 °C) accompanied by cough or sore throat, and who underwent influenza RT-PCR testing. Demographic information, including sex and age, together with specimen collection date and laboratory test results, was obtained from surveillance records. To ensure data quality and reliability, surveillance data were routinely reviewed and verified by trained public health staff at participating institutions. Laboratory testing procedures followed standardized RT-PCR protocols recommended by the National Influenza Center [18]. Duplicate records and inconsistent vaccination information were cross-checked using the immunization information system [19]. Only participants with complete demographic, vaccination, and laboratory testing information were included in the final analysis.

### 2.2. Vaccination Status

The influenza virus strains recommended by the World Health Organization (WHO) for the 2024–2025 Northern Hemisphere influenza season included A/Victoria/4897/2022 (H1N1)pdm09-like virus, A/Thailand/8/2022 (H3N2)-like virus, and B/Austria/1359417/2021 (B/Victoria lineage)-like virus [20]. In China, three influenza vaccine formulations are available: trivalent inactivated influenza vaccine (IIV3), quadrivalent inactivated influenza vaccine (IIV4), and trivalent live attenuated influenza vaccine (LAIV3), with LAIV3 licensed for children aged 3–17 years only [19]. Vaccination information, including vaccine type and administration date, was extracted from the immunization information system using each participant’s unique personal identification number. According to local vaccination practices in Wuhan, vaccination during the current season was defined as receipt of an influenza vaccine between 1 July 2024 and 31 March 2025, while vaccination during the previous season was defined as receipt of an influenza vaccine between 1 July 2023 and 30 June 2024. To evaluate the effect of vaccination history on VE, participants were classified into four categories: unvaccinated, vaccinated in both the 2023–2024 and 2024–2025 seasons, vaccinated only in the 2023–2024 season, and vaccinated only in the 2024–2025 season. Individuals were considered vaccinated if the influenza vaccine was administered at least 14 days before healthcare visit. Vaccinations administered within 14 days before the visit were excluded.

### 2.3. Statistical Analysis

Vaccine effectiveness was evaluated using a test-negative case–control design. Characteristics of RT-PCR-confirmed influenza cases were compared with those of RT-PCR-negative controls. Analyses were stratified by influenza virus type, vaccine formulation, and age group. Differences between groups were assessed using Pearson’s chi-square test, with statistical significance defined as *p* < 0.05. Adjusted VE estimates were calculated from odds ratios derived from conditional logistic regression models and expressed as (1 − odds ratio) × 100%. Separate models were fitted for each subgroup and adjusted for potential confounders, including age and sex. VE estimates were considered statistically significant when the lower limit of the 95% confidence interval exceeded zero. The test-negative design helps reduce potential bias associated with healthcare-seeking behavior because both cases and controls seek medical care for similar symptoms [8,16,21]. Additionally, restricting enrollment to patients meeting standardized ILI criteria and adjusting for age and sex further minimized confounding. All statistical analyses were conducted using R software (version 4.5.1; R Foundation for Statistical Computing, Vienna, Austria).

## 3. Results

### 3.1. Baseline Characteristics

Overall, 122,726 patients presenting with influenza-like illness (ILI) were enrolled in the analysis, including 23,302 (19.0%) RT-PCR-confirmed influenza cases and 99,424 (81.0%) RT-PCR-negative controls. Among the confirmed cases, 99.2% (23,126/23,302) were from tertiary hospitals, while 0.8% (176/23,302) were from secondary hospitals. Male patients accounted for a slightly smaller proportion (50.6%) compared to controls (52.1%). Regarding age distribution, 32.2% of cases were aged 19–59 years, and 22.1% were aged ≥70 years. The overall influenza vaccination rate was 5.93% (7273/122,726), with a vaccination rate of 3.7% (851/23,302) in the case group and 6.5% (6422/99,424) in the control group. In terms of vaccine types, the IIV4 vaccination rate in the case group was 3.3% (764/23,302), and the LAIV3 vaccination rate was 0.4% (87/23,302), whereas no IIV3 vaccinations were observed in the case group (0/23,302). In the control group, the IIV3 vaccination rate was 0.001% (6/99,424), the IIV4 vaccination rate was 5.92% (5881/99,424), and the LAIV3 vaccination rate was 0.5% (535/99,424). With the exception of vaccine type, all between-group differences were statistically significant (Table 1).

### 3.2. Weekly Distribution of Influenza Detection

**Figure 1:**
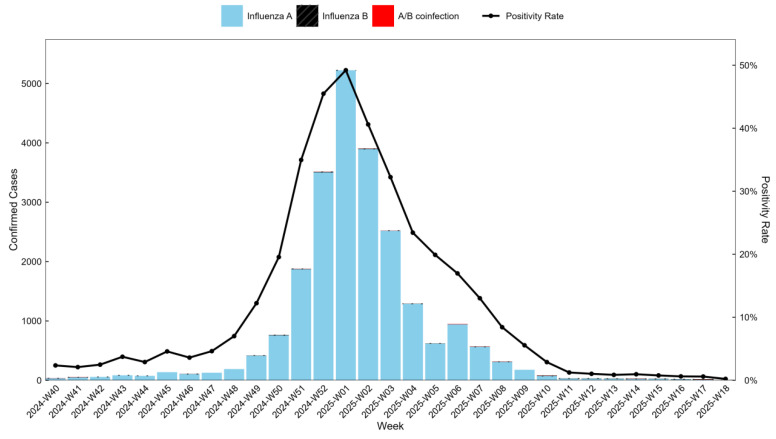
Weekly distribution of laboratory-confirmed influenza cases and test positivity rate in Wuhan during the 2024–2025 influenza season. Bars represent the number of laboratory-confirmed influenza cases by subtype, and the line indicates the weekly influenza test positivity rate.

Among RT-PCR-confirmed influenza cases, influenza A was the predominant virus type, accounting for 99.51% (23,187/23,302), whereas influenza B represented 0.33% (76/23,302) of cases. A/B co-infections were infrequently detected, comprising 0.17% (39/23,302). Both the absolute number of laboratory-confirmed influenza cases and the corresponding test positivity rate followed similar temporal patterns, increasing steadily and reaching a maximum during the first epidemiological week of 2025, when the positivity rate peaked at 49.21%. Subsequently, both indicators declined, and by week 13 of 2025, the positivity rate had decreased to 0.86%, suggesting that influenza activity in Wuhan had largely subsided (Figure 1).

### 3.3. Influenza Vaccine Effectiveness

#### 3.3.1. VE by Age Group, Vaccine Type, and Influenza Subtype

**Figure 2:**
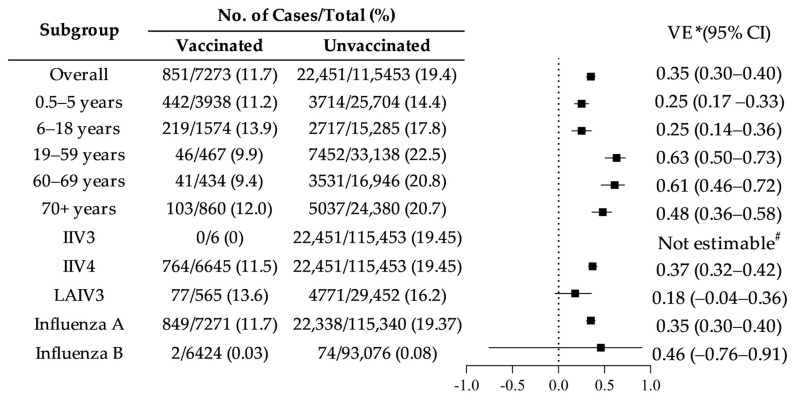
Vaccine effectiveness estimates across subgroups (* adjusted for age and sex). Error bars indicate 95% confidence intervals. Estimates with confidence intervals crossing zero are not statistically significant. ^#^ Not estimable because of insufficient sample size.

The adjusted overall VE for the 2024–2025 influenza season was estimated at 35% (95% CI: 30.1–39.8%). When stratified by age group, lower VE estimates were observed among children aged 0.5–5 years (25%, 95% CI: 17–33%) and 6–18 years (25%, 95% CI: 14–36%). In contrast, higher VE estimates were found among adults aged 19–59 years (63%, 95% CI: 50–73%) and 60–69 years (61%, 95% CI: 46–72%). With respect to vaccine formulation, IIV4 demonstrated an estimated VE of 37% (95% CI: 32–42%). Due to the extremely limited number of individuals vaccinated with IIV3 (n = 6 among controls and none among cases), VE for IIV3 could not be reliably estimated. The estimated VE for LAIV3, assessed among individuals aged 3–17 years, was 18% (95% CI: −4% to 36%), and did not reach statistical significance. When stratified by influenza virus subtype, VE against influenza A was estimated at 35% (95% CI: 30–40%). The corresponding VE estimate against influenza B was 46% (95% CI: −76% to 91%); however, this result was not statistically significant and should be interpreted cautiously given the small number of influenza B cases (Figure 2).

#### 3.3.2. VE by Influenza Season and Vaccination Month

**Figure 3:**
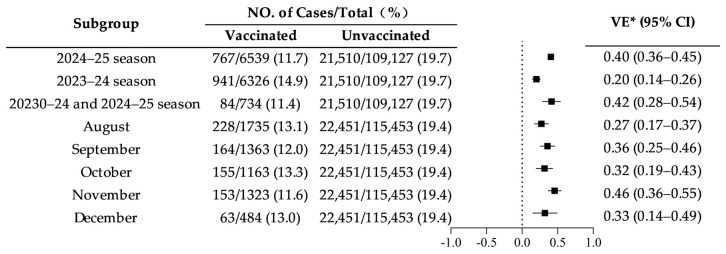
Vaccine effectiveness stratified by vaccination status in the current (2024/25) and previous (2023/24) seasons and by vaccination month (* adjusted for age and sex). Error bars represent 95% confidence intervals. Estimates with confidence intervals crossing zero are not statistically significant.

VE estimates varied according to vaccination history across influenza seasons. The highest VE was observed among individuals vaccinated in both the 2023–2024 and 2024–2025 seasons (42%, 95% CI: 28–54%), followed by those vaccinated in the current season only (40%, 95% CI: 36–45%). In contrast, VE was lowest among individuals vaccinated only during the previous season (20%, 95% CI: 14–26%). When stratified by month of vaccination, the highest VE estimate was observed for vaccination administered in November (46%, 95% CI: 36–55%), whereas the lowest VE was observed for vaccination in August (27%, 95% CI: 17–37%) (Figure 3).

## 4. Discussion

This study provides the first assessment of influenza vaccine effectiveness (VE) in Wuhan using a test-negative case–control design. The influenza vaccination coverage in Wuhan during the 2024/25 season was 5.9%, which remains relatively low compared with vaccination uptake reported in other settings. For example, national influenza vaccination coverage in China was 3.16% in the 2020/21 season and 2.47% in the 2021/22 season, whereas vaccination coverage among individuals aged ≥6 months in the United States reached 49.3% during the 2022/23 season [22]. Differences in vaccination coverage may be influenced by variations in public health promotion strategies, accessibility of vaccination services, and public perception of influenza vaccination, which historically has received less emphasis in China.

The overall adjusted VE observed in Wuhan during the 2024–2025 season was 35%, which was lower than estimates reported in Chongqing during 2018–2022 (44.4%) [23] and Hangzhou during the 2023–2024 season (48%) [24], but was comparable to interim VE estimates reported in 16 European countries for the 2022/23 season (28–46%) [11]. Age-specific analyses demonstrated higher VE among adults aged 19–59 years (63%, 95% CI: 50–73%), which may be associated with stronger immune responsiveness and fewer underlying health conditions compared with younger and older populations [12]. In contrast, lower VE estimates were observed among children aged 0.5–5 years (25%, 95% CI: 17–33%) and adolescents aged 6–18 years (25%, 95% CI: 14–36%). Similar findings have been reported in previous studies conducted in China, suggesting that relatively immature immune responses in younger populations may contribute to reduced vaccine-induced protection [25,26]. VE among adults aged 60–69 years (60.7%, 95% CI: 46–72%) remained substantial, emphasizing the importance of maintaining high vaccination uptake in older populations who are at elevated risk for severe influenza outcomes [27]. These findings are consistent with recommendations from previous studies advocating prioritization of influenza vaccination among older adults and young children [13].

In the present study, VE differed according to vaccine formulation. IIV4 provided moderate and statistically significant protection, whereas the estimated VE for LAIV3 was lower and not statistically significant. These observations are consistent with earlier reports indicating that inactivated influenza vaccines often demonstrate more stable effectiveness across different populations [28], whereas the performance of live attenuated vaccines may vary depending on host factors, pre-existing immunity, and circulating virus characteristics. Interpretation of LAIV3 results in this study should be cautious because LAIV3 was administered exclusively to children and adolescents, and the number of vaccinated individuals was limited [19]. Similar variability in LAIV effectiveness has been documented in previous seasons, particularly during periods dominated by influenza A virus circulation [29]. Furthermore, VE for IIV3 could not be reliably estimated because only a very small number of individuals received this vaccine, preventing meaningful evaluation of its protective effect.

VE also varied by influenza virus subtype. Statistically significant protection was observed against influenza A viruses, whereas VE against influenza B viruses was not statistically significant. The lack of statistical significance for influenza B is likely related to the extremely low circulation of influenza B viruses during the 2024–2025 season in Wuhan, which resulted in limited statistical precision and wide confidence intervals. Previous research has shown that VE estimates against influenza B are strongly influenced by virus circulation intensity and by the degree of match between vaccine strains and circulating lineages [9,30]. When influenza B activity is minimal, VE estimates are typically unstable and require cautious interpretation.

Vaccination history also influenced VE estimates. Individuals vaccinated in both consecutive seasons or in the current season alone demonstrated higher levels of protection compared with unvaccinated individuals. These findings support national recommendations promoting annual influenza vaccination [19]. The higher VE observed among individuals vaccinated in consecutive seasons may reflect cumulative immunological protection. However, some studies have suggested that repeated annual influenza vaccination may not always result in additive immune responses and, in certain contexts, could modify immune memory responses following repeated antigen exposure [29]. Influenza epidemics in northern China typically peak during winter and spring, whereas southern regions may experience an additional summer peak [13,19]. This seasonal pattern partly explains the extended vaccination period implemented in Wuhan. In this study, VE was highest among individuals vaccinated in November (46%, 95% CI: 36–55%) and lowest among those vaccinated in August (27%, 95% CI: 17–37%). These findings suggest that while early vaccine availability supports programmatic implementation, optimizing vaccination timing may further improve vaccine performance. Additional studies are warranted to determine the optimal vaccination schedule for maximizing protection.

The findings of this study have important implications for influenza prevention strategies in large metropolitan settings. Evidence from Wuhan suggests that optimizing vaccination timing and promoting consecutive seasonal vaccination may improve overall vaccine performance [13,29]. These findings may support refinement of local vaccination campaigns, including targeted immunization programs for high-risk populations and enhanced public health communication strategies to improve vaccine uptake [12,27]. Furthermore, integrating VE monitoring into routine influenza surveillance systems may facilitate timely evaluation of vaccine performance and support data-driven adjustment of vaccination policies [9,11].

Overall, influenza vaccination provided measurable protection against influenza infection among the population of Wuhan during the 2024–2025 season, particularly among adults. Strengthening influenza vaccination programs and increasing vaccination coverage, especially among children and adolescents, may further enhance population-level protection. In addition, promoting timely annual vaccination may improve overall vaccine effectiveness.

Limitations: The interpretation of VE estimates for certain subgroups in this study is limited by small sample size and low virus circulation. Sparse data, particularly for IIV3-vaccinated individuals and influenza B cases, can lead to unstable effect estimates and extremely wide confidence intervals, reducing the reliability of VE assessment. In particular, the extremely small number of participants vaccinated with IIV3 precluded reliable estimation of its effectiveness. Similarly, the VE estimate for LAIV3 and influenza B did not reach statistical significance, likely reflecting limited statistical power and low virus circulation during the study period. Such methodological challenges are well recognized in test-negative design studies, where low vaccine uptake or limited virus detection may lead to imprecise VE estimates [8,16]. Therefore, although subgroup analyses provide useful descriptive information, conclusions drawn from these estimates should be interpreted cautiously. In addition, this study did not include genetic subtyping of circulating influenza A strains (e.g., A(H1N1)pdm09 and A(H3N2)), which may have masked potential differences in strain-specific VE. Furthermore, the lack of detailed patient information, including residential address, chronic disease status, date of symptom onset, and antiviral drug use, may have resulted in residual confounding and affected the accuracy of VE estimates. Future studies incorporating molecular surveillance and long-term vaccination follow-up are warranted to further improve VE estimation accuracy.

## 5. Conclusions

In conclusion, this study provides region-specific evidence that influenza vaccination conferred measurable protection against laboratory-confirmed influenza during the 2024–2025 season in Wuhan. Using a test-negative case–control design, we demonstrated that overall vaccine effectiveness was moderate and varied across age groups, vaccination history, vaccine formulation, and timing of vaccination. Higher protection was observed among adults aged 19–69 years, individuals vaccinated in consecutive seasons or during the current season, and those vaccinated in November.

These findings directly address the objective of this study by quantifying influenza vaccine effectiveness in a large metropolitan setting and identifying factors associated with differential protection. The results support continued promotion of annual influenza vaccination and emphasize the importance of optimizing vaccination timing to enhance population-level protection.

## Figures and Tables

**Table 1:** Demographic characteristics of influenza test-positive and test-negative patients.

Characteristics	Cases [n (%)]	Controls [n (%)]	Total	*X* ^2^	*p*
Total	23,302 (19.0)	99,424 (81.0)	122,726		
Hospital					
Secondary	176 (0.8)	1527 (1.5)	1703	84.050	<0.001
Tertiary	23,126 (99.2)	97,897 (98.5)	121,023		
Sex					
Male	11,789 (50.6)	51,846 (52.1)	63,635	18.201	<0.001
Female	11,513 (49.4)	47,578 (47.9)	59,091		
Age group					
0.5–5	4156 (17.8)	25,486 (25.6)	29,642	802.765	<0.001
6–18	2936 (12.6)	13,923 (14)	16,859		
19–59	7498 (32.2)	26,107 (26.3)	33,605		
60–69	3572 (15.3)	13,808 (13.9)	17,380		
70+	5140 (22.1)	20,100 (20.2)	25,240		
Vaccinated in 2024/2025 season					
Yes	851 (3.7)	6422 (6.5)	7273	266.327	<0.001
No	22,451 (96.3)	93,002 (93.5)	115,453		
Types of Vaccines					
IIV3	0	6 (0.001)	6	4.208	0.122
IIV4	764 (3.3)	5881 (5.92)	6645		
LAIV3	87 (0.4)	535 (0.54)	622		

## Data Availability

The data presented in this study are not publicly available due to institutional restrictions on data sharing, as the data were derived from the hospital information system (HIS). Access to the data may be granted by the corresponding author upon reasonable request and subject to approval by the data-owning institution.

## Abbreviations

The following abbreviations are used in this manuscript:

VE	Vaccine Effectiveness
ILI	Influenza-like illness
RT-PCR	Real-time reverse transcription polymerase chain reaction
OR	Odds ratio
CI	Confidence interval
IIV3	Trivalent inactivated influenza vaccine
IIV4	Quadrivalent inactivated influenza vaccine
LAIV3	Trivalent live attenuated influenza vaccine

## Appendix A

**Table A1:** Source Hospitals and Number of Cases.

Hospital Level	Hospital Name	Number Tested by RT-PCR
Secondary		1703
	Wuhan No. 9 Hospital	969
	Hubei RongJun Hospital	655
	Wuhan East Lake Hospital	55
	Wuhan Red Cross Hospital	24
Tertiary		121,023
	Wuhan Pulmonary Hospital	382
	Hubei Government Hospital	239
	The First People’s Hospital Of Jiangxia District, Wuhan City	160
	People’s Liberation Army Airborne Corps Hospital	17
	Hospital of Wuhan University of Science and Technology	17
	Wuhan Children’s Hospital	14,223
	The Central Hospital Of Wuhan	13,135
	Wuhan NO. 1 Hospital	12,812
	Maternal and Child Health Hospital Of Hubei Province	10,753
	Union Hospital, Tongji Medical College, Huazhong University of Science and Technology	8699
	Tongji Hospital, Tongji Medical College, Huazhong University of Science and Technology	6496
	Wuhan Puren Hospital	6195
	The Fifth Hospital Of Wuhan	5543
	The Forth Hospital Of Wuhan	4350
	China Resources & Wuhan Iron and Steel General Hospital	3775
	Zhongnan Hospital Of Wuhan University	3476
	The Third People’s Hospital Of Hubei Province	2635
	Tianyou Hospital Affiliated To Wuhan University Of Science & Technology	2003
	Wuhan Third Hospital	1821
	Hubei Provincial Hospital of TCM	1418
	Wuhan Jinyintan Hospital	852
	General Hospital of Central Theater Command	696
	Wuhan Hospital Of Traditional Chinese Medicine	504
	Hubei General Hospital	118
	Hubei Cancer Hospital	2
	Wuhan Caidian District People’s Hospital	3325
	Wuhan Asia General Hospital	2778
	Taikang Tongji (Wuhan) Hospital	2176
	The Eight Hospital Of Wuhan	1445
	Wuhan Hankou Hospital	1120
	Wuhan Taikang Hospital	59
	Geriatric Hospital Affiliated to Wuhan University of Science and Technology	8
	Wuhan Huangpi District People’s Hospital	6075
	Wuhan Dongxihu District People’s Hospital	2681
	The Sixth Hospital Of Wuhan	606
	THE SECOND HOSPITAL OF WISCO	384
	Wuhan Hanyang Hospital	45
Total		122,726
